# Isolated sphenoid sinus foreign body following slingshot injury: a case report

**DOI:** 10.1186/s13256-026-06142-4

**Published:** 2026-06-09

**Authors:** Brendan Luu, Craig Mooney, Rataphol Chris Dhepnorrarat, Pav Gounder

**Affiliations:** 1 Department of Ophthalmology, Sir Charles Gairdner Hospital, Hospital Avenue, Nedlands, Western Australia Australia; 2 Centre for Ophthalmology and Visual Science, University of Western Australia, Perth, Western Australia Australia; 3 Department of Otolaryngology Head Neck Skull Base Surgery, Sir Charles Gairdner Hospital, Perth, Western Australia Australia; 4 University of Western Australia, Perth, Western Australia Australia

**Keywords:** Slingshot injury, Sphenoid sinus, Foreign body, Trauma, Orbit

## Abstract

**Background:**

Impaction of foreign bodies within the paranasal sinuses secondary to slingshot projectiles is uncommon, with only three documented case reports within the literature. There are no clear guidelines for the management of these injuries, and given the heterogeneity of their presentations, various methods of foreign body extraction have been employed, ranging from enucleation of the affected eye to transnasal endoscopic approaches.

**Case presentation:**

This case report describes the presentation and successful management of a 36-year-old male with a ruptured left globe and no perception of light (NPL) visual acuity secondary to a slingshot projectile. CT imaging of the head and orbits revealed a 17 mm-diameter metallic foreign body impacted solely within the sphenoid sinus. A review of the literature confirmed this to be the first reported case of an isolated sphenoid sinus foreign body secondary to a slingshot projectile mechanism.

**Conclusions:**

This report highlights the necessity of a multidisciplinary approach in managing complex sino-orbital foreign body injuries. It underscores the ophthalmic rationale and benefits of primary repair of a globe perforation in the setting of NPL vision, as well as the novel endoscopic techniques employed to achieve minimally invasive foreign body retrieval.

## Introduction

Slingshot-induced paranasal sinus foreign bodies (FBs) are rare, with only three reported cases in the literature [1–3]. In instances of penetrating facial and orbital trauma, the sphenoid sinuses are uncommonly involved, with the orbit, frontal, and maxillary sinuses more likely to be injured [4]. This report details the ophthalmic management of a penetrating globe rupture and the novel endoscopic approach utilised to retrieve an isolated sphenoid sinus FB secondary to a slingshot projectile injury.

## Case report

A 36-year-old male was transferred to our metropolitan tertiary hospital emergency department (ED) in Perth, Western Australia from a remote ED via the Royal Flying Doctor Service following an alleged assault to his left eye with a slingshot and metal projectile. The patient was otherwise fit and well, with no significant past medical, family, or psychosocial history. Initial assessment upon presentation to the remote ED showed that he was haemodynamically stable with a Glasgow Coma Score of 15/15. Ophthalmic examination of the left eye revealed no perception of light (NPL) visual acuity (VA), marked periorbital swelling, hyphaema, and a horizontal corneal laceration with extruding intraocular contents. Right eye VA was 6/6 (20/20). These findings were confirmed by the ophthalmology team upon presentation to our metropolitan ED. Given the extensive periorbital swelling and extruding intraocular contents, relative afferent pupillary defect and posterior segment examination could not be performed. Cranial nerve and extraocular motility assessments were also limited by periorbital swelling and pain.

**Fig. 1:**
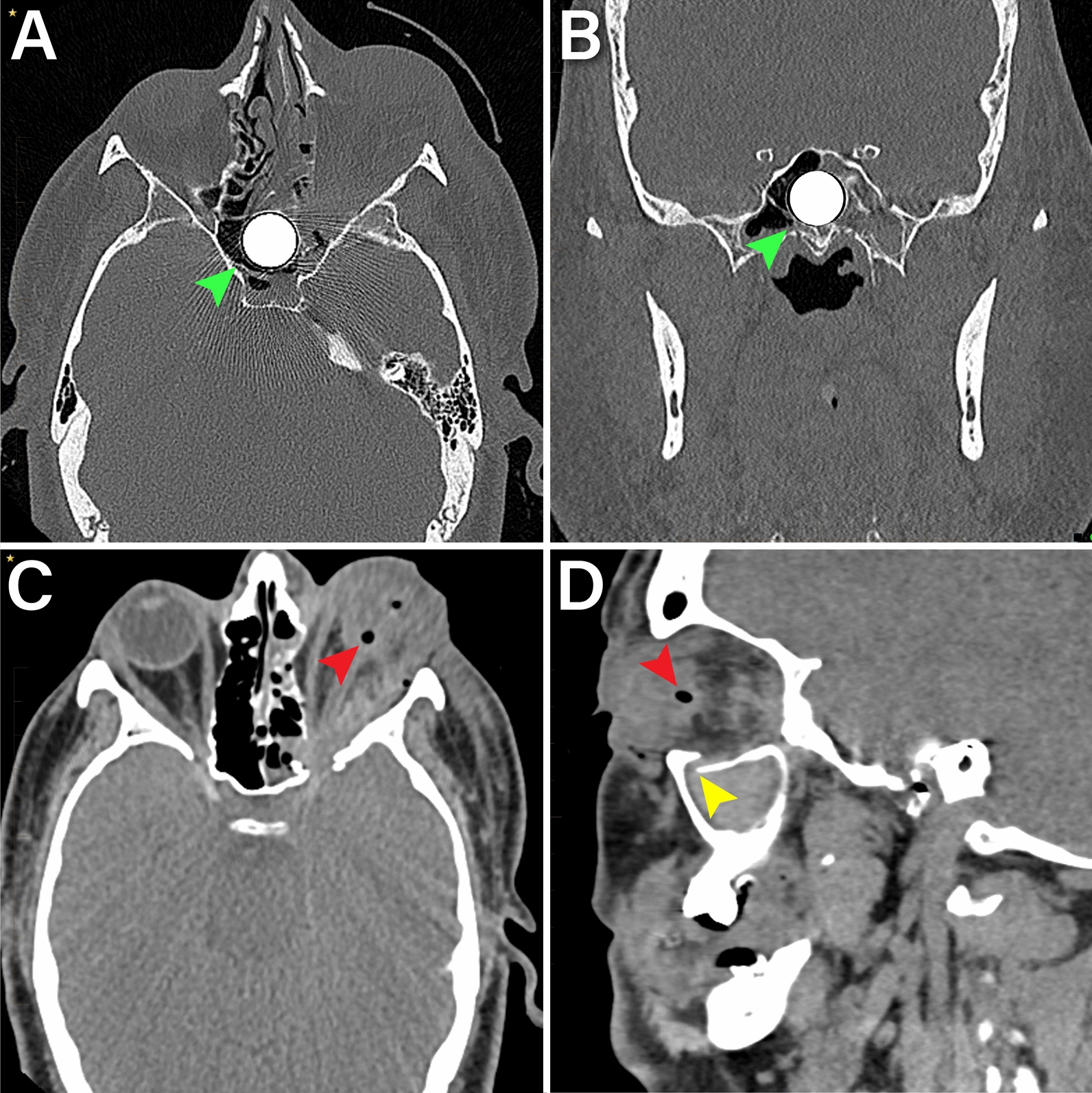
CT head and orbits depicting the impacted foreign metallic body in the sphenoid sinus. Bony windows in the axial (**A**) and coronal planes (**B**) reveal a 17 × 17 mm hyperdense foreign body (green arrows) lodged in the sphenoid sinus. Soft tissue windows show evidence of globe rupture with associated intraocular and intraorbital gas (red arrows) in the axial and sagittal plane (**C**, **D**). An orbital floor fracture (yellow arrow) is seen in the sagittal plane (**D**)

As shown in Fig. 1, an emergency CT of the head and orbits demonstrated complete rupture of the left globe, with a blowout orbital floor fracture and a 17 mm-diameter impacted metallic foreign body (FB) within the sphenoid sinus. There were fractures of the medial orbital wall and the lateral margins of the superior orbital fissure with significant distortion of the left ethmoid and maxillary sinuses. Visualised intracranial contents were normal with no evidence of a skull base fracture, pneumocephalus, or haemorrhage.

Following discussion with otorhinolaryngology (ENT) colleagues, the patient underwent urgent combined ophthalmic and ENT surgery shortly after presentation to our tertiary centre. Examination under general anaesthesia revealed a 180-degree horizontal, full-thickness scleral/corneal laceration with prolapsed uveal tissue, which was excised. Muscle insertions were unable to be identified. Scleral and then conjunctival tissues were sutured closed.

Endoscopic sinus surgery was then performed to remove the metallic FB. Examination of the left nasal cavity revealed significant oedema and trauma, with a normal nasal passage on the right side. The CT scan revealed a sphenoid inter-sinus septum fracture with displacement into the right sphenoid sinus, with the FB occupying a midline position. A right sided approach to the FB was then performed to avoid the significantly distorted and traumatised left side. A maxillary antrostomy with complete sphenoethmoidectomy facilitated a combined transnasal/transethmoid approach to the right sphenoid sinus. The sphenoidotomy was then expanded to identify the fractured inter-sinus septum, which was removed to reveal the FB. A partial posterosuperior septectomy was performed to improve access, and a left sphenoidotomy was completed. Removing the smooth, heavy FB proved difficult with conventional sinus surgery equipment and a magnetised Nasogastric bridle retrieval probe (Bridle™, First™ Medical Company) was used to remove it from the sinus cavity. As the FB exceeded the width of the nasal cavity on either side, a partial posterior septectomy with backbiting instruments was required to widen the posterior choana, allowing displacement of the FB into the post-nasal space and subsequent trans-oral delivery. The sphenoid sinus was then thoroughly irrigated and suctioned to remove any organic matter transferred from the FB. This also allowed for careful inspection for injury to the optic nerves, paracavernous carotid arteries, and pituitary sella. The cavity was also inspected for a cerebrospinal fluid leak, which was not present.

Post-operative X-ray confirmed successful removal of the metal FB and bilateral opacification of the maxillary sinuses was also noted, consistent with expected post-surgical changes. The patient recovered uneventfully with no acute complications and was discharged 3 days post-operatively with NPL vision in the left eye, and 6/6 VA in the right. At the 2-month post-operative ophthalmic review, the left eye maintained NPL vision and had progressed to phthisis bulbi with marked reduction in orbital volume. The patient was subsequently referred to an ocularist for consideration of a prosthetic eye. At the 4-month ENT follow-up, there was no evidence of residual sinonasal pathology, and the patient was discharged from further ENT care.

## Discussion

Given the deep and central location of the sphenoid sinus within the skull base, it is uncommon to see retained FBs in this location. A 2002 retrospective review of all patients presenting with isolated sphenoid sinus disease over a 25 year period at a single Shanghai University Hospital described only 8 cases of sphenoid FBs out of a total of 122 patients [5].

Slingshot-type projectiles are capable of generating sufficient kinetic energy to impart moderate to critical craniofacial trauma, yet penetrating orbital injury from this mechanism is exceptionally rare, with globe-perforating slingshot injuries representing only 2 of 72 perforating ocular injuries in one Australian series, and only three case reports in the literature describe the impaction of a FB within the paranasal sinuses secondary to this mechanism [1–3, 6]. To our knowledge, this is the first case report in the literature that describes an isolated sphenoid sinus FB in an adult or paediatric patient, secondary to a slingshot projectile through the orbit. This conclusion is based on a systematic search of PubMed, MEDLINE, and Google Scholar conducted in April 2026, using the terms “sphenoid sinus”, “foreign body”, “slingshot”, “paranasal sinus”, “isolated”, and “projectile” with no language or date restrictions, which yielded no comparable case.

Projectile-related mechanisms account for a significant proportion of open globe injuries presenting to tertiary centres, representing 34.1% of open globe repairs in one large series, and are the most common mechanism of open globe injury in men [7, 8]. While these injuries are relatively well-characterised in the literature, the specific subset, wherein a slingshot projectile traverses the orbit to impact within the paranasal sinuses remains sparsely documented. In these cases, variations in projectile trajectory, entry point, and location of the impacted FB account for the heterogeneity in presentations and subsequent management strategies. Mendoza et al. reported an 11-year-old girl with a ruptured left globe and NPL vision, with a glass marble abutting the left sphenoid and posterior ethmoid, managed by enucleation and endoscopy-assisted transorbital FB removal [1]. Vinuthinee and colleagues described an 11-month-old child who sustained a slingshot injury to the left orbit with globe-sparing injury and retained frontal bone FB, managed by direct surgical debridement and FB removal via the open wound [2]. More recently, Lai et al. reported a metallic FB in the left posterior ethmoid sinus following a slingshot strike to the left medial upper eyelid, with globe integrity confirmed intraoperatively and FB retrieval achieved via a combined transnasal endoscopic approach [3].

The trajectory of the FB in this case can be inferred from the clinical and radiological findings. The projectile likely entered through the anterior cornea, as evidenced by the horizontal corneal laceration, and traversed the orbit in a posterior and slightly inferior direction along the orbital axis, as demonstrated on the sagittal CT (Fig. 1D). The force of impact fractured the medial orbital wall and orbital floor, with the blowout fracture suggesting inferiorly directed force transmission, before the projectile continued posteriorly to impact within the sphenoid sinus. The midline position of the FB on axial imaging (Fig. 1A, B) further supports a medial trajectory. The optic canal and superior orbital fissure, both formed by the sphenoid bone, represent natural conduits through which penetrating FBs may access the deeper skull base, and the superior orbital fracture raises the possibility of transit through or immediately adjacent to these structures [9].

In cases of facial and orbital trauma, the paranasal sinuses can act as “crumple zones” to protect more delicate intracranial structures from injury [10, 11]. The complete sparing of intracranial contents and absence of pneumocephalus on CT highlights this protective role in the present case. The sequential fracturing of the medial orbital wall and orbital floor, the bony boundaries of the ethmoid and maxillary sinuses respectively, likely dissipated sufficient projectile energy to arrest the FB within the sphenoid sinus before it could reach the skull base vasculature or brain parenchyma [12].

Visual prognosis following globe injury is largely predicted by the severity of the presenting VA, with lower initial VA and more posterior injuries being associated with poor outcomes [13, 14]. Secondary complications, such as proliferative vitreoretinopathy and endophthalmitis, are also associated with further deterioration [15]. The Ocular Trauma Score (OTS) provides a validated framework for prognostication in open globe injuries, and the combination of presenting NPL vision and globe rupture alone placed the patient in the lowest OTS category, which carries the highest probability of NPL outcome [15, 16]. The progression to phthisis bulbi at 2 months is also consistent with the expected natural history of a severely disrupted globe, reflecting hypotony-driven atrophy following loss of structural integrity [17]. As phthisical eyes have no visual potential, management is directed toward cosmetic rehabilitation and symptom relief, consistent with our decision to refer the patient to an ocularist for prosthetic rehabilitation [18].

Given their low incidence and lack of standardised protocols, the management of these injuries often warrants multidisciplinary approaches between ophthalmic, ENT, and/or neurosurgical colleagues. The decision to primarily repair the ruptured globe reflects the paradigmatic shift in the management of open globe injuries presenting with NPL vision. Historically, these eyes were managed with primary enucleation to mitigate the risk of sympathetic ophthalmia; however, emerging data has challenged this understanding, with a recent systematic review and meta-analysis by Patterson et al. reporting no statistically significant reduction in risk of sympathetic ophthalmia following primary enucleation [19, 20]. Although not reflected in the outcome of our case, primary repair also offers the, albeit limited, prospect for some degree of visual rehabilitation given advances in vitreoretinal surgical techniques [21, 22]. From a cosmetic vantage point, in place globes also allow future ocular prosthetics to maintain mobility compared to silicone orbital implants [23]. Regarding the impacted FB, endoscopic sinus surgery offers a low morbidity approach for the retrieval of sinonasal FBs with excellent visualisation, no external incisions, and minimal pain [24]. True intra-sinus FBs are rare but standard and extended sinonasal approaches offer access to all areas of the sinonasal cavity. The size and location of the FB dictates the approach, and as demonstrated in this case, retrieval may require novel instrumentation, even in instances with excellent access and visualisation.

## Conclusion

Given the rare and heterogenous nature of sino-orbital injuires secondary to projectile mechanisms, a collaborative multidisciplinary approach is recommended to optimise diagnosis, management, and outcomes. Notably, this case sheds light on the visual and functional benefits of primary repair over enucleation in the management of NPL-presenting globe ruptures. Additionally, it highlights the novel endoscopic techniques that may be employed by surgeons in the retrieval of isolated sphenoid sinus FBs.

## Data Availability

You may contact the corresponding author for further case information if required.

## Abbreviations

NPL: No perception of light
CT: Computed tomography
FB: Foreign body
ENT: Ear, nose, and throat (otorhinolaryngology)
